# Degradation of Typical Reverse Sand-Mudstone Interbedded Bank Slope Based on Multi-Source Field Experiments

**DOI:** 10.3390/ijerph20032591

**Published:** 2023-01-31

**Authors:** Zhenwei Dai, Luqi Wang, Xiaolin Fu, Bolin Huang, Senlin Zhang, Xuecheng Gao, Xiangrong He

**Affiliations:** 1 Wuhan Center, China Geological Survey (Central South China Innovation Center for Geosciences), Wuhan 430205, China; 2 School of Civil Engineering, Chongqing University, Chongqing 400045, China; 3 Hubei Key Laboratory of Disaster Prevention and Mitigation, China Three Gorges University, Yichang 443002, China

**Keywords:** evolution process, the Three Gorges Reservoir Area, field test, rocky bank, reverse sand-mudstone interbed slope

## Abstract

The bank slopes in the Three Gorges Reservoir area (TGRA) have experienced obvious deterioration under the action of the periodic fluctuations in the reservoir water level. Generally, laboratory tests have been used to reveal the evolution trend of the slope banks. However, this method has a certain degree of cross-scale problem, especially for the mechanical state in a complex environment. Therefore, in this study, we took the Yangjiaping bank slope in the TGRA as an example and proposed a comprehensive on-site detection method to further reveal the rock mass degradation phenomenon of this typical reverse sand-mudstone interbedded bank slope. Specifically, multi-scale laser scanning, cross-hole acoustic wave detection, and inclination measurements were performed to analyze the fractures, quality, and deformation of rocky banks. The results showed that the deterioration of the bank slope manifested as the expansion, deepening, and widening of the cracks, as well as the peeling off and loosening of rocky banks. Large-scale laser scanning revealed that the deterioration zone was deformed along large fracture zones and layers. Unlike limestone slopes, the intact sandstone underground might be degraded by changes in water. There are few inclinometers and no deformation or weak deformation, which requires long-term monitoring. The relevant research methods provide an important reference for determining the instability and failure trend of the reservoir bank slopes.

## 1. Introduction

As one of the most important hydropower stations in the world, in addition to hydropower generation and water transportation, the Three Gorges Reservoir area (TGRA) is particularly important for regional sustainable water management [1,2]. Since 2008, the Three Gorges Dam has been operated experimentally at a water level of 175 m, and the reservoir water level has fluctuated periodically between 145 and 175 m every year [3,4]. These changes in the reservoir water level have occurred for more than 10 years, causing damage to the rocky banks in the reservoir area [5,6,7]. This deterioration is not only reflected by the extension and expansion of fractures, but also by the weakening of the mechanical parameters of the reservoir bank rock mass [8,9], such as the cohesion and internal friction angle. In this case, the bank slopes have evolved toward the unstable stage, especially in the Wuxia Gorge, Xiling Gorge, and Qutang Gorge, which are composed of nearly 500 km long karst bank slopes. For example, the damage to the Jianchuandong rock mass, Banbiyan rock mass, Guanmuling rock mass, and Longmenzhai rock mass may lead to collapse and surge disasters, endangering the safety of the Yangtze River waterway [10,11,12]. Therefore, the rock mass deterioration of karst bank slopes has been of great concern and has become one of the key problems in geological disaster prevention and mitigation in the TGRA [13,14,15]. Notably, the collapses of rocky banks are characterized by gradual deformation and sudden destruction, which increases the difficulty of determining the deterioration process and failure trend.

Regarding the changes in the rock and soil mass caused by the reservoir water level, the evolution trend was analyzed. Based on long-term monitoring data, Yin et al. [16,17] determined the evolution trend of the reservoir banks under the effect of the reservoir water level, and a simplified calculation model was developed to analyze the evolution trend of the reservoir bank in a complex mechanical environment. Various indoor test methods and theoretical derivations were used to analyze the deterioration trend of the high and steep reservoir banks under hydraulic coupling [10,18]. After considering the test results under dry–wet cycles, the finite element numerical calculation method was applied to study the evolution trends of the reservoir bank slopes [7,19]. Feng et al. [20] analyzed the dam slopes of several major hydropower stations in China (e.g., the Jinping I, Xiaowan, Baihetan, Xiluodu, Wudongde, Laxiwa, Ertan, and Dagangshan stations). They proposed that faults and discontinuities should be emphasized in studying rocky slope stability.

Existing studies have proposed the possible mechanism, phenomenon, and change trend of the rock strength of corroded rock masses in the fluctuation zone of the reservoir water level [21,22,23]. Specifically, cyclic weathering caused by the reservoir water level affects the mechanical properties of rock mass at the macroscopic level and intact rock properties at the microscopic level [24,25,26,27]. However, there is still a large knowledge gap in determining the deterioration degree of such rocky banks, which is the basis for analyzing and judging the long-term stability of bank slopes [28,29,30].

**Figure 1:**
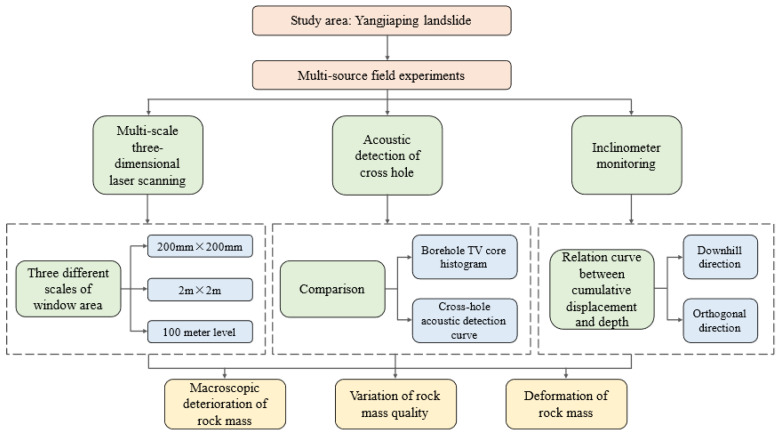
The degradation analysis methods of the typical reverse sand–mudstone interbedded bank slope based on multi-source field experiments.

To better understand the deterioration of rocky banks under variations in reservoir water level, in this study, a method for comprehensively investigating typical reverse sand–mudstone interbedded bank slopes was developed based on multi-source field experiments (Figure 1). The results of this study provide basic technical support for the prevention and control of damage to rocky reservoir slopes around the world.

## 2. Study Area

**Figure 2:**
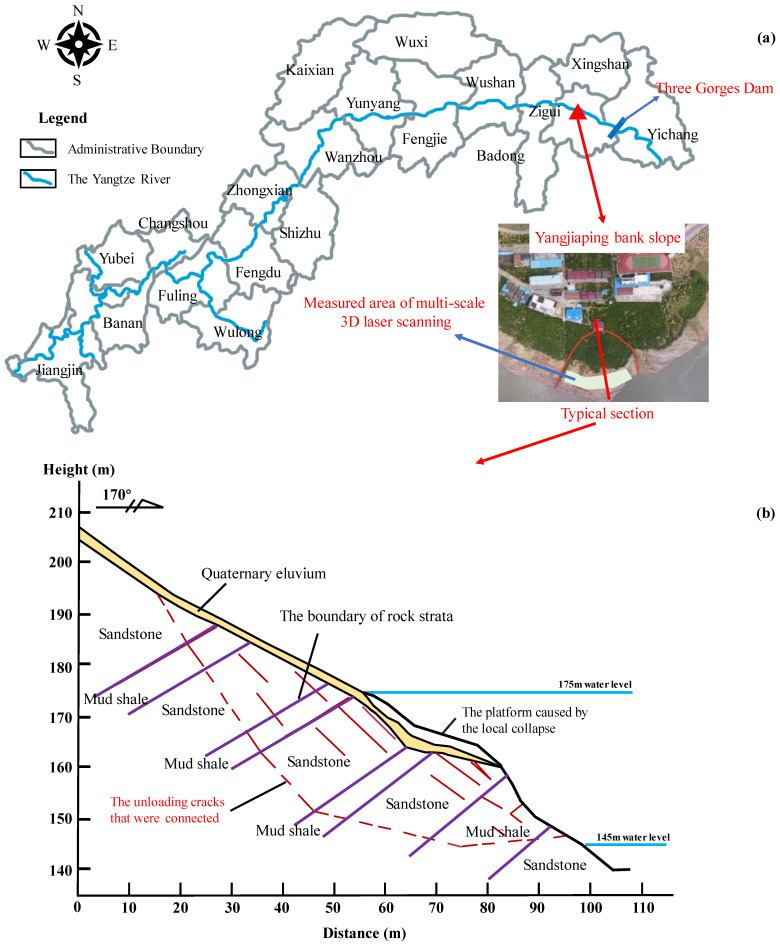
The (**a**) location, and (**b**) typical section of the Yangjiaping bank slope.

In this study, the Yangjiaping bank slope, a typical reverse sand–mudstone interbedded bank slope, was selected for a case study. This slope is located in Zigui County, Hubei Province, China (Figure 2a). The Yangjiaping bank slope is composed of clastic rocks of the upper Triassic Shazhenxi Formation and the lower Jurassic Xiangxi Formation, and the strata are characterized by alternating soft and hard rocks. The hard rocks in the slope are mainly sandstone and siltstone, and the soft rocks are weak silty mudstone and mud shale. Under the action of the periodic fluctuations in the reservoir water level, the rock masses on the slope are easily disintegrated and broken, and the physical mechanical properties of the rock mass deteriorate significantly. In this study area, the soft rocks deform, and the hard rocks are pressed down. Specifically, the sandstone undergoes bending deformation similar to that of a cantilever beam under the joint action of its weight and the vertical load. During the bending deformation process, the bank slope slides along the bedding layer and rotates toward the free surface, resulting in the formation of tensile cracks perpendicular to the bedding layer. Moreover, the original gently inclined structural surface is pulled apart and expanded, and the deformation gradually develops from the surface downward and from the top to the foot of the slope. When the unloading cracks gradually connect the slope foot to a certain position, the bending deformation reaches the critical state, and the slope-rock mass becomes unstable as a whole (Figure 2b).

## 3. Multi-Scale Three-Dimensional (3D) Laser Scanning

Various scales of laser scanning were used to build a 3D surface cloud model [31,32]. The typical deterioration mode of the slope surface was obtained by comparing multiple periods. The measurement scales were 100 m, 2 m, and 0.2 m. Furthermore, the measured data from two hydrological years were analyzed to study the deterioration trend. The scanned areas were mainly distributed in the deterioration zone, that is, the area where the reservoir water level rose and fell periodically. Specifically, for the measured area with a smaller scale, several types of joints were selected for comparative analysis. When the scale of the measurement area was expanded, the joints were also used as the key information for capturing and selecting the specific area.

### 3.1. The Measurement Scale of 0.2 m

**Figure 3:**
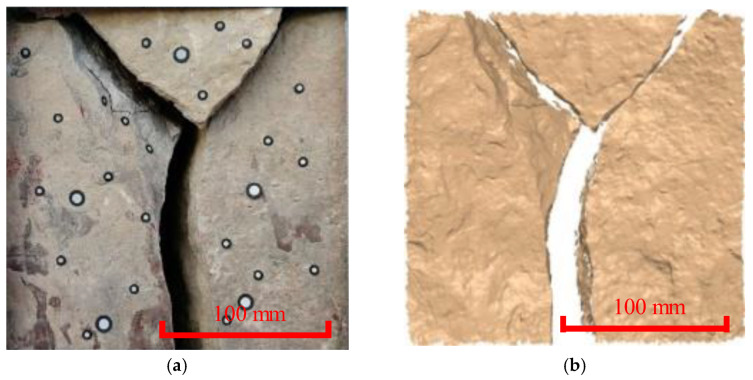

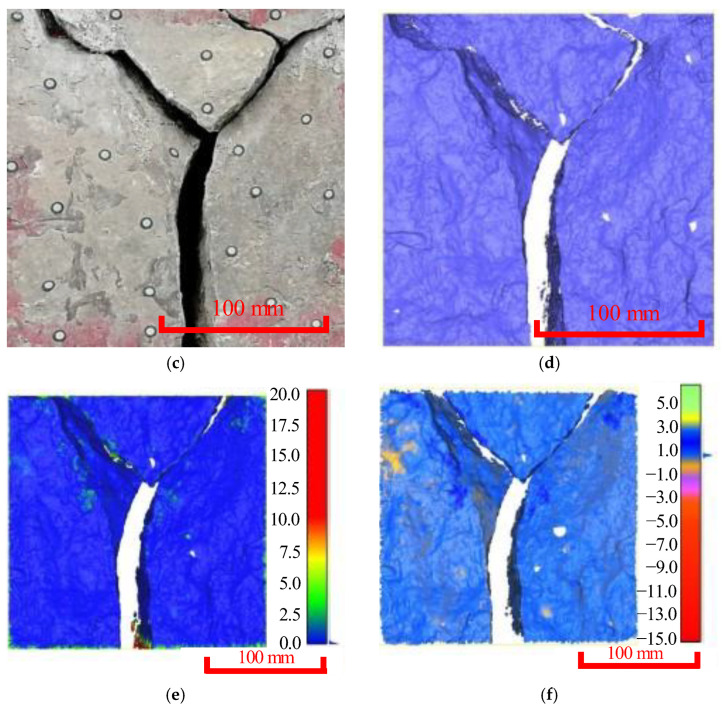
In situ and 3D cloud diagram of Y-joint. (**a**) Site picture of the measuring area (Period I). (**b**) Image of the 3D laser scanning (Period I). (**c**) Site picture of the measuring area (Period II). (**d**) Image of the 3D laser scanning (Period II). (**e**) Deformation distribution obtained using the C2C method. (**f**) Deformation distribution obtained using the M3C2 method.

The Y-shaped joints, oblique joints, dense joints, vertical joints, and weathered areas were studied separately. Based on the comparison of the cloud diagram of the Y-shaped joints (Figure 3), there was a Y-shaped joint in the middle, and there was no obvious change during the two-year monitoring campaign. Direct cloud-to-cloud comparisons using the closest point technique (C2C) and multi-scale model-to-model cloud comparison (M3C2) were used to quantify the deformation trends. Specifically, the C2C is not sensitive to small deformations, while the M3C2 can capture the small deformation more accurately. These two algorithms revealed that the maximum deformation of the two phases was approximately 1 mm. Based on the pairing and point cloud error, it was found that there is currently no substantial deterioration and deformation in the survey area. However, it is predicted that the upper rock mass may fall off and slip along the joint surface due to the cutting of wide joints. The reservoir water periodically soaks along the Y-shaped joint, the rock mass softens, and its stability is greatly reduced.

**Figure 4:**
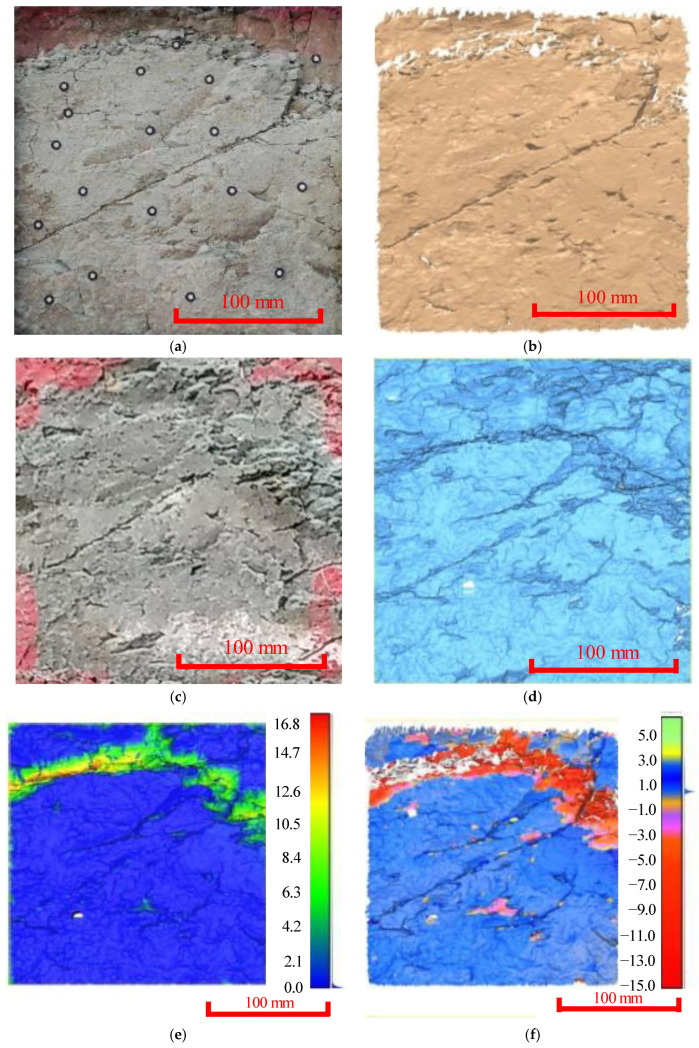
In situ and 3D cloud diagram of oblique joints and peeling-off blocks. (**a**) Site picture of the measuring area (Period I). (**b**) Image of the 3D laser scanning (Period I). (**c**) Site picture of the measuring area (Period II). (**d**) Image of the 3D laser scanning (Period II). (**e**) Deformation distribution obtained using the C2C method. (**f**) Deformation distribution obtained using the M3C2 method.

Regarding the oblique joint cutting the rock surface in the middle (Figure 4), the upper rock mass was fragmented and broken during Period II, with local falling off and obvious erosion and spalling along the oblique joints. According to the analysis results obtained using C2C and M3C2 methods, the deformation of this area was characterized by inclined joint collapse, denudation and block falling, and the formation of wider cracks. It was found in Figure 4e,f that the accuracy was sub-millimeter level, and the amount of erosion of the joint was 5–15 mm. The results of these two algorithms were also compared at the same location, and the maximum difference was approximately 1 mm. Thus, the adopted algorithms could finely depict and capture the widening and deepening of the joints and the rock peeling.

**Figure 5:**
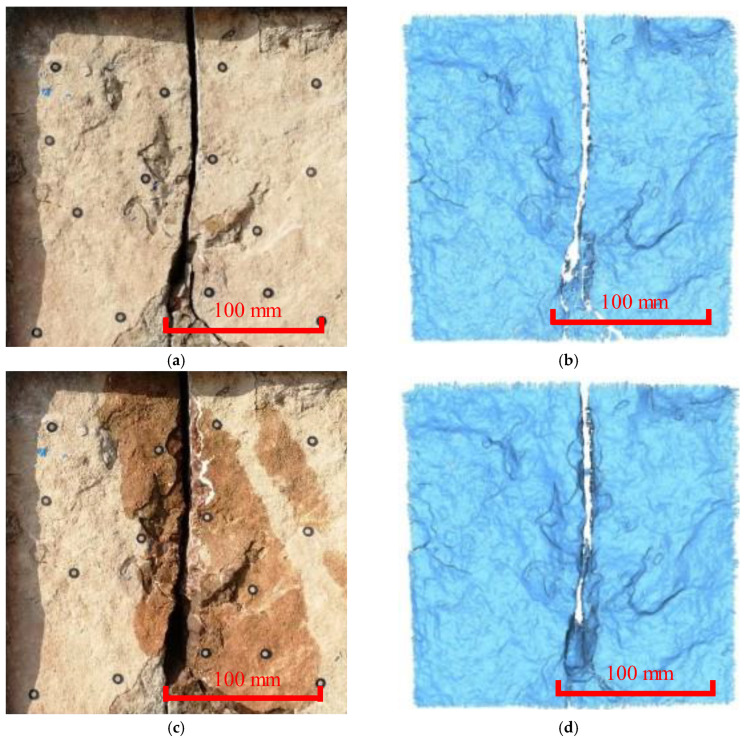

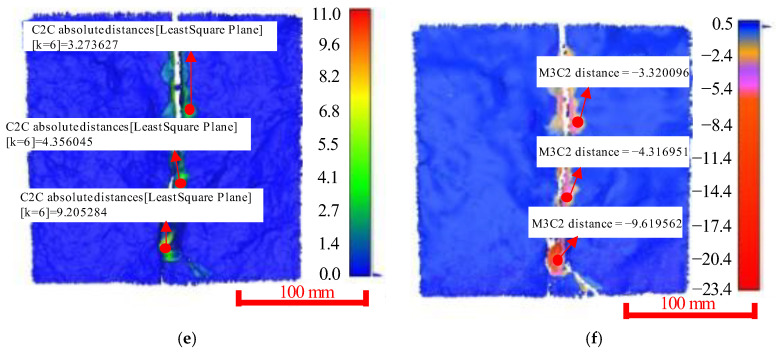
In situ and 3D cloud diagram of vertical joints. (**a**) Site picture of the measuring area (Period I). (**b**) Image of the 3D laser scanning (Period I). (**c**) Site picture of the measuring area (Period II). (**d**) Image of the 3D laser scanning (Period II). (**e**) Deformation distribution obtained using the C2C method. (**f**) Deformation distribution obtained using the M3C2 method.

By comparing the cloud data and site pictures, it was found that the middle vertical joint experienced widening and erosion (Figure 5). Both sides of the middle joint experienced erosion and widening of the cracks. According to Figure 5e,f, the amount of erosion of the crack was 3.27–9.62 mm, the crack width increased from 7 mm to 15 mm, and the widest part was approximately 25 mm. It was concluded that when the crack widened, both sides of the crack “underwent erosion and extension,” exhibiting a hollow-strip shape. Since the crack in the middle was long and narrow, the deformation sign of the point cloud was strip-shaped with a blank area in the middle and deepened erosion on both sides.

**Figure 6:**
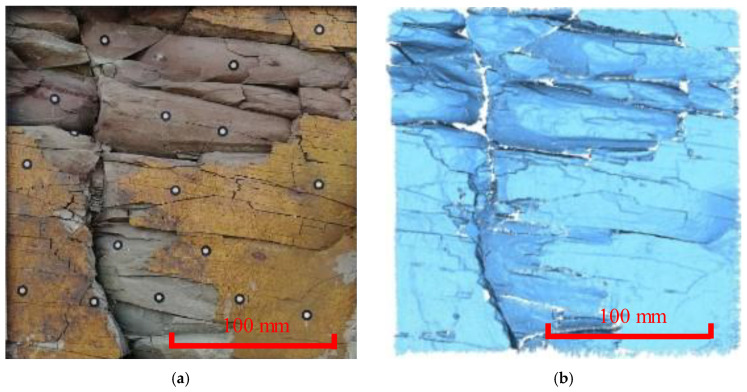

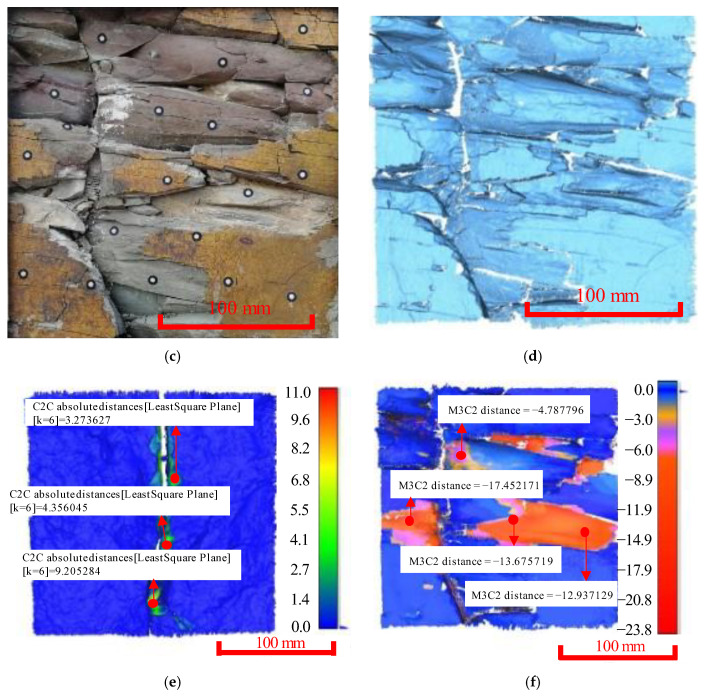
In situ and 3D cloud diagram of the dense joint area. (**a**) Site picture of the measuring area (Period I). (**b**) Image of the 3D laser scanning (Period I). (**c**) Site picture of the measuring area (Period II). (**d**) Image of the 3D laser scanning (Period II). (**e**) Deformation distribution obtained using the C2C method. (**f**) Deformation distribution obtained using the M3C2 method.

By comparing the cloud data and site pictures of the dense joint area (Figure 6), it was found that flaky spalling occurred, and the joints were corroded and widened under the fluctuation of the reservoir water level. As can be seen from Figure 6e,f, the amount of erosion of the crack was 4.78–17.45 mm. When the block experienced spalling, the deformation characteristics of the cloud data indicated irregular flaky erosion. This phenomenon differs from the striped point cloud response of the manifestation and expansion type of the crack described above. The boundary of the corresponding deformation was the position of the falling block.

**Figure 7:**
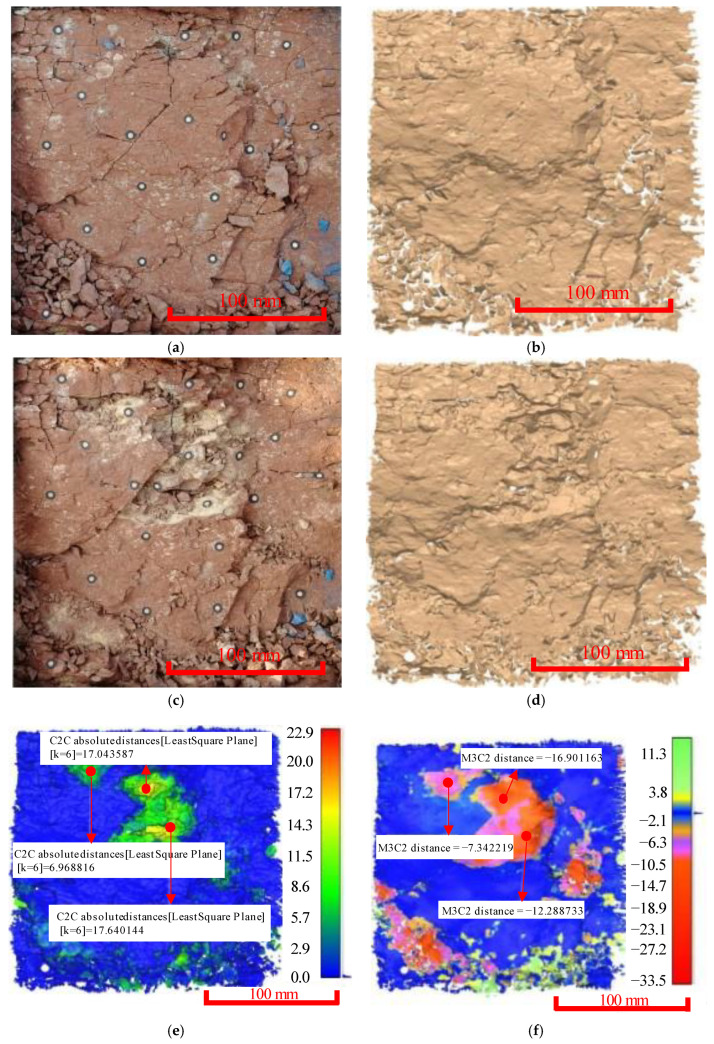
In situ and 3D cloud diagram of the weathered area. (**a**) Site picture of the measuring area (Period I). (**b**) Image of the 3D laser scanning (Period I). (**c**) Site picture of the measuring area (Period II). (**d**) Image of the 3D laser scanning (Period II). (**e**) Deformation distribution obtained using the C2C method. (**f**) Deformation distribution obtained using the M3C2 method.

Regarding the deformation trend of the weathered area (Figure 7), there were many flaky peeling blocks, and some of the cracks were eroded and widened. As can be seen from Figure 6e,f, the amount of erosion of the cracks was 6.97–17.64 mm. By comparing the results for the same position, it was found that the difference in the absolute displacement between these two algorithms was approximately 0.37 mm, and only one recorded point exhibited a large deviation of approximately 5.35 mm due to the sparse point cloud data and the large fluctuation in the rock surface. When the blocks were peeling and falling, the point cloud deformation exhibited signs of irregular flaky erosion, and the corresponding deformation boundary was the position of the block peeling off.

### 3.2. The Mesurement Scale of 2 m

**Figure 8:**
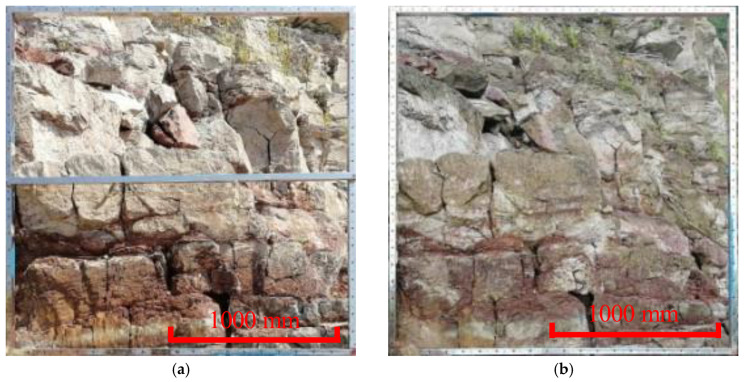
In situ pictures of measured area (2 m × 2 m). (**a**) Site picture of the measuring area (17 August 2019). (**b**) Site picture of the measuring area (12 July 2020).

**Figure 9:**
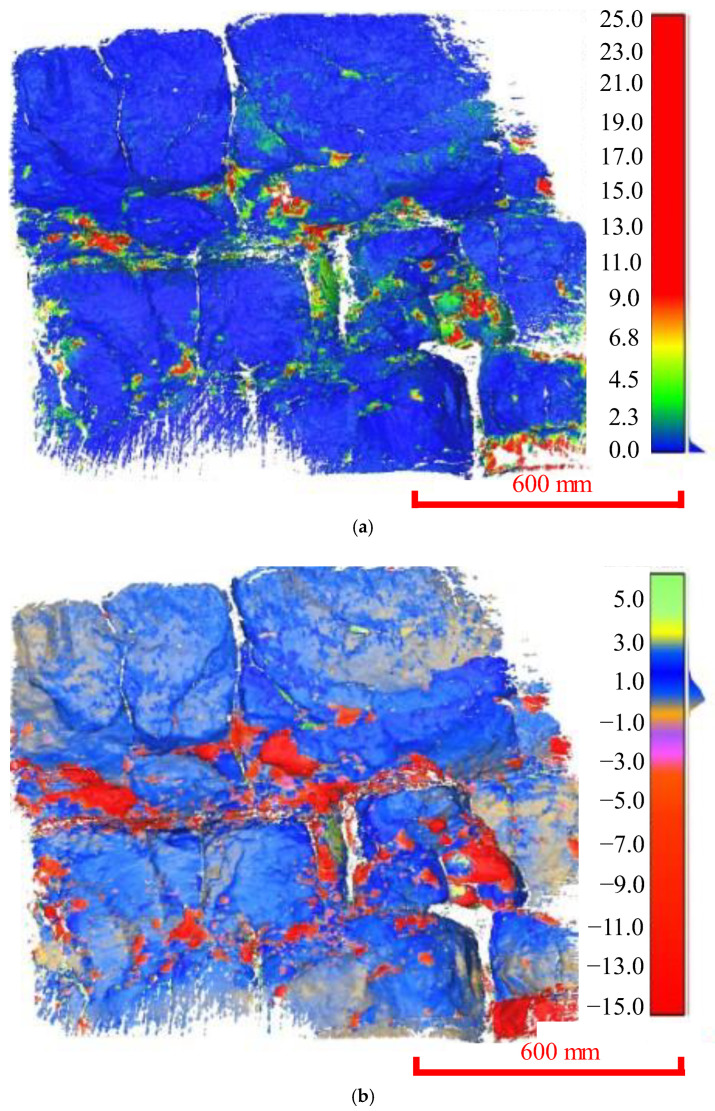
The 3D cloud diagrams of the measured area (2 m × 2 m). (**a**) Deformation distribution obtained using the C2C method. (**b**) Deformation distribution obtained using the M3C2 method.

Aiming at the widely developed cutting fractures and fragmented thinly bedded rock surfaces in the Yangjiaping bank slope, a typical 2 m × 2 m area was selected for 3D laser scanning (Figure 8). After comparing the 3D laser scanning cloud data for the different periods (Figure 9), we obtained the joint expansion, extension, rock surface fragmentation, and other degradation information (e.g., crack depth, crack extension, and crack shape).

Based on the in situ pictures and 3D laser scanning, the distribution of the joints, cracks, and rock layers had an important impact on the deterioration of the rock mass. Specifically, the deterioration of the banks accelerated along the joints and cracks. Due to the cutting of the discontinuities, the mechanical properties of the rock mass decreased sharply. Under the action of the stress concentration and the fluctuations in the reservoir water level, it was easy for the cracks to extend farther along the tips of the cracks. For the rock mass divided by cracks, the influence of the fluctuation in the reservoir water level was more obvious.

The regional deformation mainly occurred in the steep hills and the places where the undulation of the joints changed greatly, forming irregular strips. The C2C method can be used to calculate the absolute value of the deformation. Still, the relative information about the rock mass, i.e., erosion versus accumulation, cannot be effectively judged using this method. The C2C method can be used to quickly and efficiently determine the main area of deformation, providing a reference for further accurate deformation calculations using the M3C2 method. Using the C2C method, it was found that the deformation was approximately 15 mm, and the main interface of deformation was 1.5 mm. Furthermore, according to the results of the M3C2 method, a similar rule was obtained, the deformation was approximately 20 mm, and the main deformation interface was 3.5 mm. According to the deformation thresholds, the 3D degradation rate can be calculated as follows:

(1) $$D=\frac{n}{N}$$

where *D* is the degradation rate; *n* is the number of points greater than the deformation threshold; and *N* is the total number of points. For the C2C method, *n* = 315,219, *N* = 2,311,638, and thus, *D* = 13.63%. Similarly, for *n* = 32,059 and *N* = 2,462,952 in the M3C2 method, *D* = 14.97%. It was found that the volume strain values calculated using these two algorithms were similar, and the mean value of the degradation rate was 14.30%.

**Figure 10:**
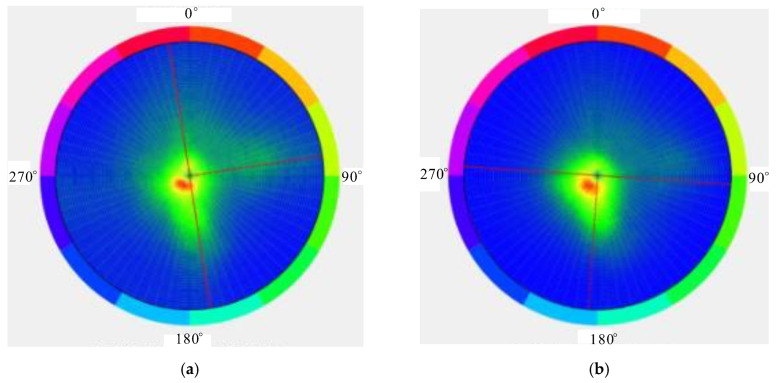
Occurrence distribution of micro-elements of Yangjiaping bank slope. (**a**) Occurrence distribution of micro-elements (17 August 2019). (**b**) Occurrence distribution of micro-elements (12 July 2020).

The occurrence of the micro-elements can be extracted via 3D laser scanning (Figure 10). The average inclination angle during Period I was approximately 0°, and the average inclination was 81°, indicating that the surfaces were relatively flat. Correspondingly, the average inclination angle during Period II was approximately 2°, and the average inclination was 183°. Due to erosion, the slope fluctuated and became rough. This change in the occurrence was consistent with the field survey. Notably, the dominant joints in the study area were still similar (8°∠220°).

**Figure 11:**
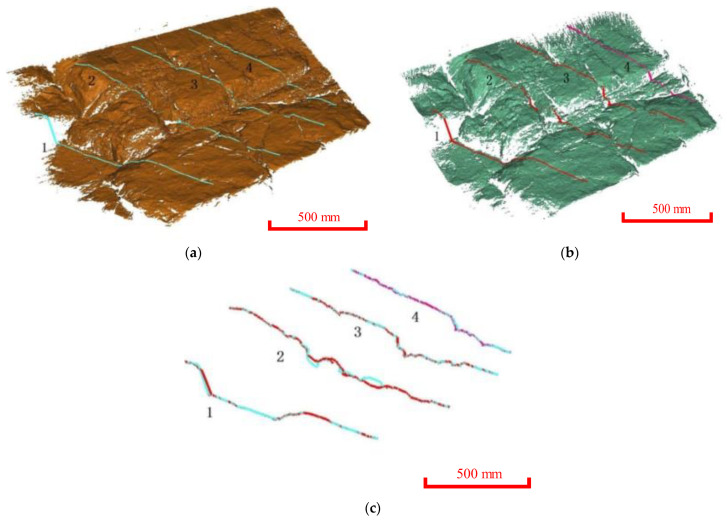
Comparison of typical sections of Yangjiaping bank slope; (**a**) The location of the typical sections (17 August 2019). (**b**) The location of the typical sections (12 July 2020). (**c**) The superposition of two-period measurement data.

Four typical sections were extracted to analyze the surface changes (Figure 11). It was found that the measured data for Period II was lower than those for Period I, indicating that the overall change was dominated by erosion and peeling. The mudstone and sandy mudstone in the study area soften and disintegrate when exposed to water, and their strength decreases sharply. With immersion under the fluctuating reservoir water level, the water gradually infiltrates into the slope, and the joints gradually deepen and widen. Under this mechanism, the rock mass will further disintegrate, causing shallow landslides.

### 3.3. The Meaurement Scale of 100 m

**Figure 12:**
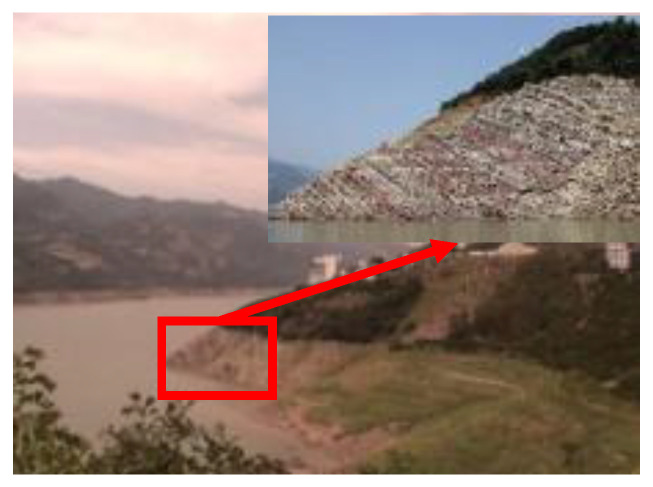
The study area with a measurement scale of 100 m.

The overall outline of the Yangjiaping bank slope was obtained using an Optech ILRIS-LR large-scale 3D laser scanner. The transmission frequency of this instrument is 10 kHz, the farthest range is 3 km, and the comprehensive accuracy is 2 cm. The 3D scans (Figure 12) were conducted on 30 August 2019, and 24 June 2020. Furthermore, the related parameters of the 3D laser scan are listed in Table 1.

**Figure 13:**
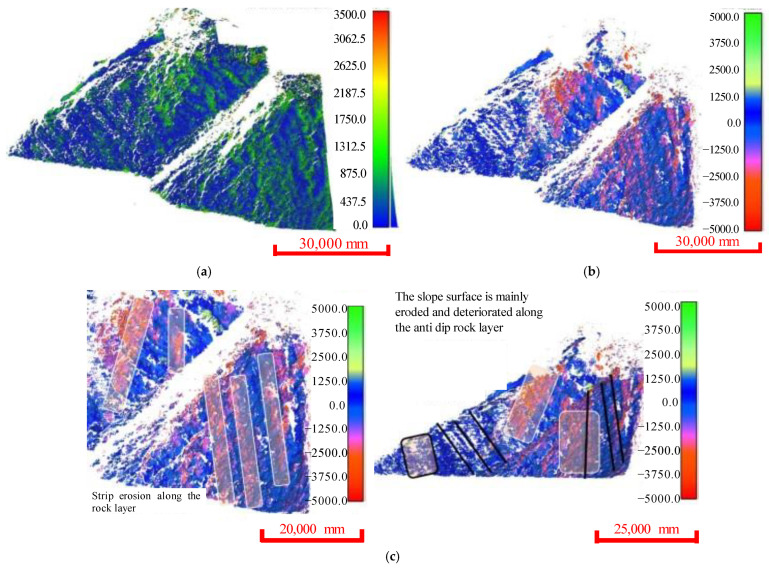
The 3D deformation of a large-scale Yangjiaping bank slope. (**a**) Deformation distribution of the measured area after C2C analysis. (**b**) Deformation distribution of the measured area after M3C2 analysis. (**c**) Details of slope deformation.

The comparison results (Figure 13) showed that the deformation mainly occurred in the scarps and rock layers, with an irregular strip distribution. As the Yangjiaping bank slope is mainly composed of soft rock, the deformation in this large area was greater than 1 m, and the area with the largest deformation was located near the rock layer. Considering that the accuracy of large 3D laser scanning was approximately 2 cm, the main deformation threshold was set to 300 mm. According to Equation (1), the 3D deterioration rates obtained using the C2C and M3C2 methods were 54.41% and 80.06%, respectively.

**Figure 14:**
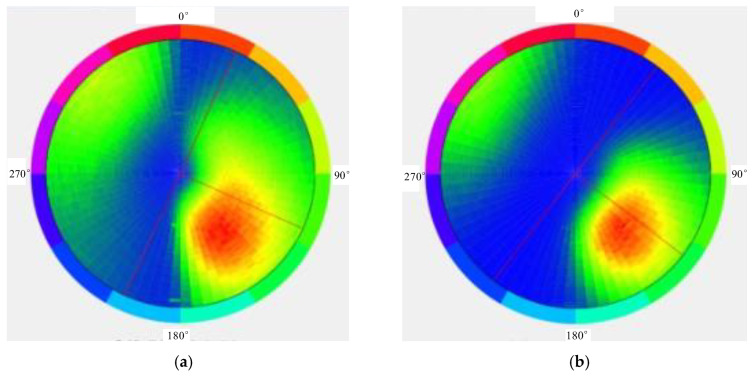
Occurrence distribution of micro-elements on a large-scale bank slope. (**a**) Occurrence distribution of micro-elements (30 August 2019). (**b**) Occurrence distribution of micro-elements (24 June 2020).

**Figure 15:**
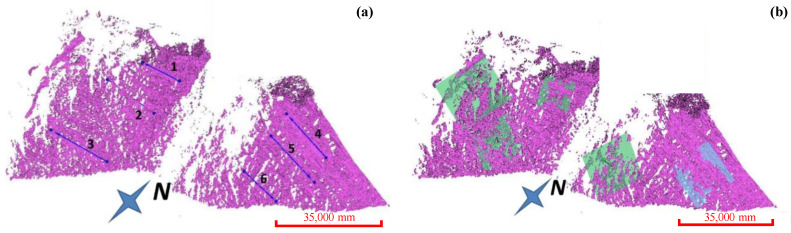
The distribution of joint traces and occurrences. (**a**) Trace measurement of joints. (**b**) Occurrence measurement of joints.

The occurrence distribution of the micro-elements was extracted from the 3D laser scanning data (Figure 14). The average dip angle of the measurement data during Period I was approximately 61°, and the average dip was 114°. The average inclination of the measurement data during Period II was approximately 65°, and the average inclination was 127°. The steepening of the dip was due to the expansion of the scanning range to the east in 2020. The occurrence of the slope obtained during different periods was approximately 63°∠120.5°, which was consistent with the field survey. Tracking and measuring the occurrences of multiple joint traces (Figure 15) revealed that there were three to four types of joint combinations that cut the bank slope into fragments, and collapses also occurred along the rock layers. Detailed information on the joint traces and occurrences is shown in Table 2.

**Figure 16:**
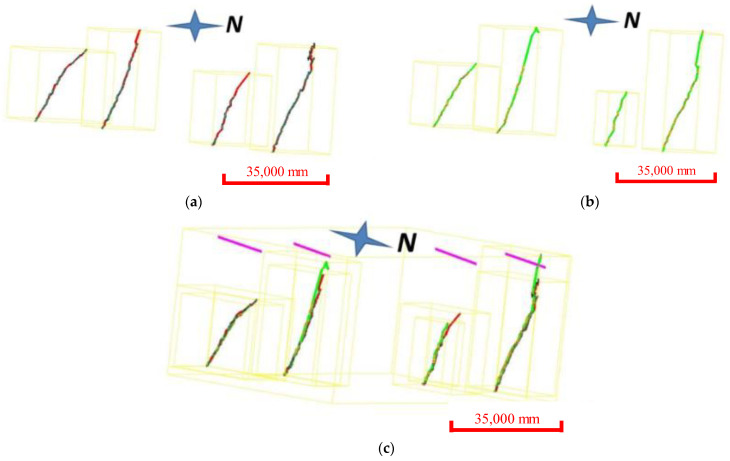
Comparison of typical cross-sections of the 3D laser scanning. (**a**) Typical sections in 2019. (**b**) Typical sections in 2020. (**c**) Comparison of typical sections.

A comparison was made among the four typical sections extracted from the point clouds for the two periods (Figure 16). The sections for the two measurement periods were mostly non-overlapping. Still, the difference in the non-overlapping area was relatively small. Most of the measurement data in the non-overlapping area obtained during Period II were lower than those obtained during Period I, indicating that the overall deterioration and deformation area of the bank slope was relatively extensive.

## 4. Acoustic Detection in Cross-Boreholes and Inclinometer Tests

The average slope of the Yangjiaping bank was 40°. Except for a few sandstone layers, most of the rock masses on the slope were broken. The acoustic detection in the cross-boreholes and the inclinometer tests were conducted at 146 m, 161 m, and 175 m.

**Figure 17:**
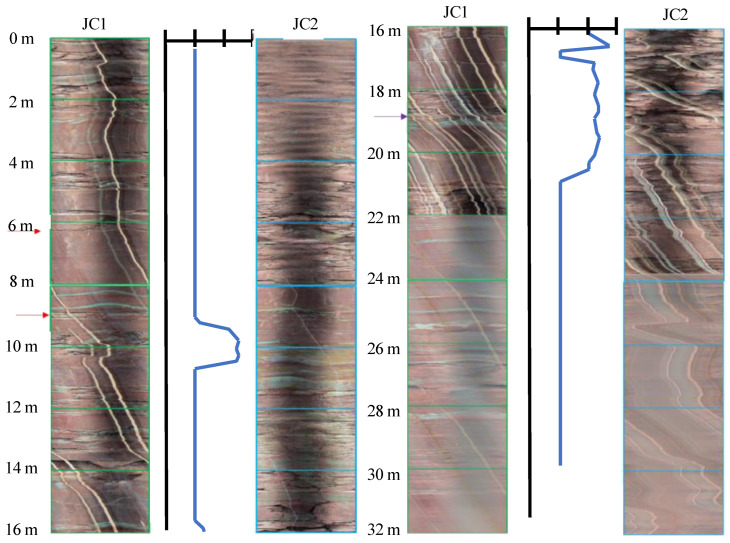
Comparison of borehole images and acoustic velocity measurement (146 m).

Figure 17 shows a comparison of JC1 and JC2 located at 146 m. According to the borehole images, the stratum was mainly purple-red mudstone within 30 m, the rock mass was broken, and macroscopic structural plane bedding was developed. When the mudstone was relatively intact, clear acoustic data were obtained, such as at depths of 8–11 m and 17 m. In the intervals where the mudstone rock mass was relatively broken or where there were many structural bedding planes, effective acoustic signals could not be received because the energy loss of the ultrasonic waves was very fast when propagating in the broken rock mass, and the wave-scattering phenomenon was relatively serious. The energy of the effective wave passing through was lower than that of the noise, so the propagation time of the wave could not be accurately obtained.

**Figure 18:**
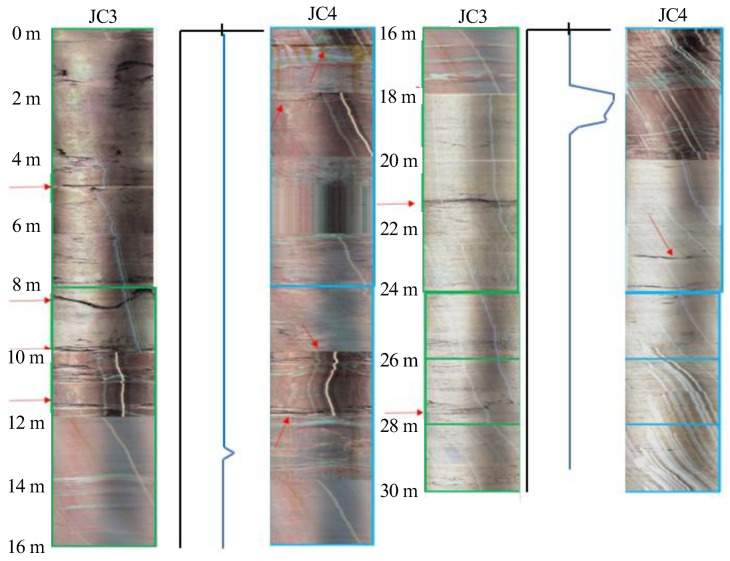
Comparison of borehole images and acoustic velocity measurement (161 m).

Figure 18 shows a comparison of JC3 and JC4 located at 161 m. Due to the higher elevation, JC3 and JC4 were less affected by the reservoir water than JC1 and JC2. There were two obvious weak interlayers near 8.1 m and 21.5 m in JC3, and the rock mass was broken at 0–8 m and 10–12 m. According to the cross-hole acoustic wave detection curves, there was an obvious effective signal near 13 m, and the wave velocity increased, indicating that the rock mass was intact at these positions. This section corresponded well with the borehole images. At a depth of 18 m, there was a significant lithological interface, and the wave velocity suddenly increased, indicating that the quality of the next section of the rock mass was high. There was an obvious weak interlayer at a depth of 21.5 m, which made it impossible to obtain valid data later.

**Figure 19:**
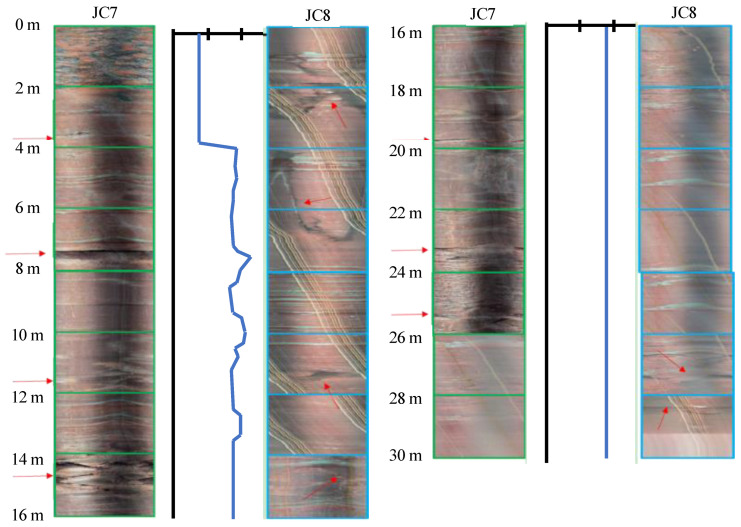
Comparison of borehole images and acoustic velocity measurement (175 m).

Figure 19 shows a comparison of JC7 and JC8 located at 175 m. From 0 to 26 m, the stratum was mainly purple-red mudstone. Specifically, there were obvious weak interlayers at approximately 8 m and 14.2 m, and the rock mass was very broken at 0–2 m and 24–26 m. The rock mass was relatively intact below 26 m. Correspondingly, the wave velocity anomalies at 8 m, 10 m, and 13 m revealed the presence of structural planes. After 14 m, no effective acoustic signal was received.

**Figure 20:**
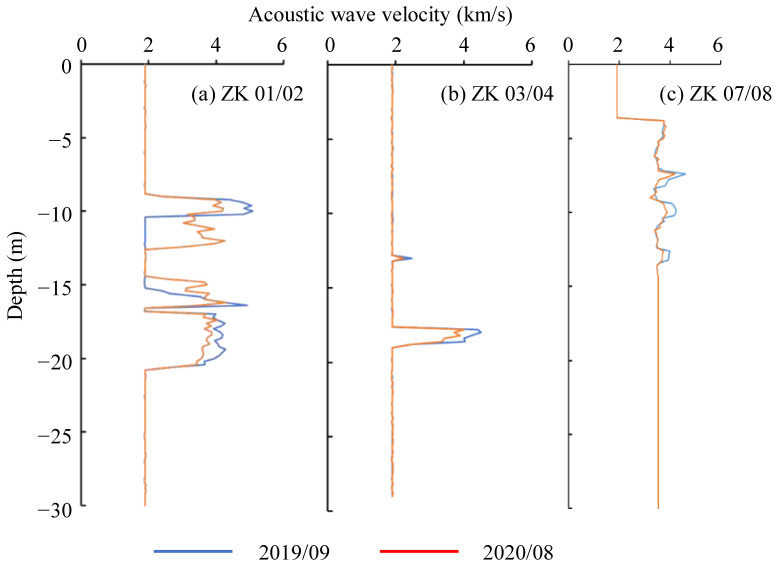
Curves of the cross-borehole acoustic wave velocity changing with depth.

The curves of the changes in the cross-borehole acoustic wave velocity with depths at 146 m, 161 m, and 175 m were obtained (Figure 20). Notably, the ZK is the abbreviation of the specific boreholes for the test of cross-hole acoustic wave velocity, and JC is the abbreviation of the specific boreholes for the inclinometer monitoring. Figure 20a shows that in the depth intervals 8.8–10.6 m and 15–21 m, the effective cross-hole acoustic wave values were 5.09 km/s and 4.03 km/s, respectively. Because most of the rock masses were relatively broken, the wave values in the other depth intervals were invalid. Correspondingly, the effective values of the cross-borehole acoustic wave velocity at depths of 12.8–13.6 m and 17.6–19 m in ZK03/04 were 2.49 km/s and 4.50 km/s, respectively (Figure 20b). The effective value of the cross-borehole acoustic wave velocity was obtained in ZK07/08 at depths of 7–13.6 m was 4.42 km/s (Figure 20c).

According to the lithology and ultrasonic characteristics, the effective wave velocity region corresponded to relatively intact sandstone. The wave velocity data for Period II exhibited a certain decrease, which may have been induced by the deterioration of the rock mass. The decrease in the wave velocity of the sandstone section was analyzed (Table 3), and the average rate of decrease was approximately 7.4%.

**Figure 21:**
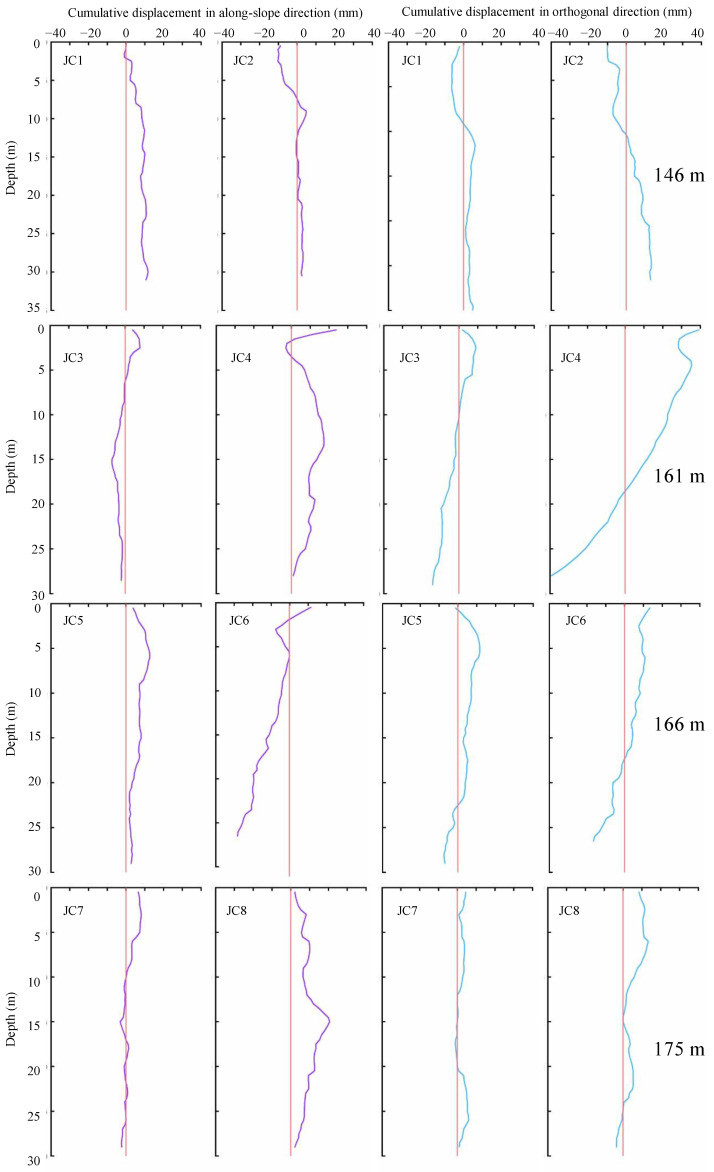
Changes in inclinometer monitoring data.

The changes in the reservoir water caused the times and cycles of the inclinometer tests to be different. To facilitate later data collection, according to the characteristics of the “cross” shape of the inclinometer borehole, two directions were labeled: the along-slope direction and the orthogonal direction. The positive and negative values of the cumulative displacement represent the forward and reverse inclinometer measurements, respectively. According to the above method, the data were collected every 0.5 m, shown in Figure 21.

Based on the comparison and analysis of the inclinometer observations during the two periods (Figure 21), there were different degrees of cumulative displacement in the along-slope direction and the orthogonal direction. The long-term effect of the changes in the changing reservoir water level caused the rock mass to deteriorate, which was manifested as softening, cracking, and crushing of the soft rock along the structural planes and rock layers [33,34]. The current inclinometer surveys may still be insufficient; thus, long-term monitoring is necessary [35,36,37].

## 5. Discussion

To comprehensively reveal the degradation of a typical reverse sand–mudstone interbedded bank slope, multi-scale 3D laser scanning, cross-hole acoustic detection, and inclinometer tests were conducted to study the macro-deterioration phenomenon under the action of water level changes. Regarding the deformation of the reservoir bank, in general, multiple single points were selected on the slope to monitor the long-term displacement. However, it is difficult for multi-point displacement to reflect the overall deformation state of a landslide, even for a dense layout based on a typical section. The methods adopted in this study overcome this limitation to a certain extent.

Due to the specific structure of the reverse sand–mudstone interbedded bank slope, the deterioration was centrally distributed in the sections affected by the water variations. This is because mudstone and sandstone are sensitive to water, unlike bank slopes composed of limestone, such as the Jianchuandong dangerous rock mass [38], Huangyanwo dangerous rock mass [6], and Banbiyan rocky bank [12]. In this study, the parameters during the two periods were compared. Although only one cycle of water level fluctuation had occurred, the weak deformation could already be captured. Continuous monitoring could obtain a more obvious deterioration trend, which would provide an important reference for the degradation of reservoir banks [39,40,41,42,43].

Moreover, laboratory tests, such as dry–wet cycle tests, can quickly simulate the deterioration during multiple phases in a short time, and combined with numerical simulations, they can also provide better quantitative results regarding the overall stability of the rock slope. As the conditions in field tests are closest to the actual mechanical state, they provide a very important reference for cross-scale analysis. Specifically, laboratory tests and numerical simulations can be further corrected and verified according to field test results [44,45]. The long-term stability trends of rocky banks can be determined more accurately by combining multi-source data obtained via various methods. By 2023, the total population in the TGRA is predicted to reach 17 million. The prevention of damage to rocky banks is of great significance for regional geological safety and is also conducive to achieving the UN sustainable development goals (SDGs).

## 6. Conclusions

Accompanied by obvious deterioration under the action of the periodic fluctuations in the reservoir water level, it is difficult to determine the degradation trend of rocky banks. In this study, the Yangjiaping bank slope in the TGRA was taken as an example, and a comprehensive on-site detection method was developed to further reveal the rock mass degradation phenomenon of this typical reverse sand–mudstone interbedded bank slope. Specifically, multi-scale laser scanning, cross-hole acoustic wave detection, and inclination measurements were performed to investigate the fractures and the quality and deformation of rocky banks.

Under the action of the reservoir water level fluctuations, the deterioration of the reverse sand–mudstone interbedded bank slopes was mainly characterized by the expansion, deepening, and widening of the cracks, as well as spalling and loosening. Through multi-scale 3D laser scanning, the deformation of the slope during different periods was captured. The results showed that the deformation was greatest in the cracks and layers. The distribution of the joints, cracks, and rock layers had an important impact on the degradation of the mechanical properties of the rock mass in the banks; that is, the degradation of the rock mass was accelerated along the discontinuities.

As the conditions in field tests are closest to the actual mechanical state, field tests provide a very important reference for cross-scale analysis. Furthermore, laboratory tests and numerical simulations can be further corrected and verified according to field test results. The long-term stability trends of rocky banks can be determined more accurately by combining multi-source data obtained using various methods.

## Figures and Tables

**Table 1:** Detailed parameters of the 3D laser scan.

Date	Rows	Cols	Number of Points	Scan Area (m^2^)
30 August 2019	4428	6995	6,120,697	5312
24 June 2020	3845	7647	5,635,568	6020

**Table 2:** Detailed information on the joint traces and occurrences.

Number of Joints Traces	Dip Angle (°)	Dip Direction (°)	Number of Joints	Dip Angle (°)	Dip Direction (°)
1	35	121	1	40	127
2	22	82	2	68	130
3	24	107	3	32	169
4	35	91	4	47	147
5	33	89	5	26	152
6	32	85	6	35	50
/	/	/	7	29	37
/	/	/	8	40	33

**Table 3:** Wave velocity of the sandstone section.

Boreholes	Depth (m)	Wave Velocity in 2019 (km/s)	Wave Velocity in 2020 (km/s)	Drop Rate (%)
ZK01/02	8.8~10.6	5.09	4.57	10.22
	15~21	4.03	3.72	7.69
ZK03/04	12.8~13.6	2.49	2.35	5.62
	17.6~19	4.50	4.22	6.23
ZK07/08	7~13.6	4.42	4.10	7.24

## Data Availability

Not applicable.
